# Fishers' knowledge and seahorse conservation in Brazil

**DOI:** 10.1186/1746-4269-1-12

**Published:** 2005-12-08

**Authors:** Ierecê ML Rosa, Rômulo RN Alves, Kallyne M Bonifácio, José S Mourão, Frederico M Osório, Tacyana PR Oliveira, Mara C Nottingham

**Affiliations:** 1 Departamento de Sistemática e Ecologia, Centro de Ciências Exatas e da Natureza, Universidade Federal da Paraíba, 58059-900 João Pessoa, PB, Brazil; 2 Departamento de Biologia, Centro de Ciências Biológicas e da Saúde, Universidade Estadual da Paraíba, 58109-753, Campina Grande, PB, Brazil; 3 Centro de Ciências Biológicas e da Saúde, Universidade Estadual da Paraíba, 58109-753, Campina Grande, PB, Brazil; 4 Departamento de Engenharia de Pesca, Laboratório de Biologia Aquática, Universidade Federal do Ceará, Av. Mister Hull, s/n, 60455-760, Fortaleza, CE, Brazil; 5 Jardim Botânico Benjamin Maranhão, Av. Dom Pedro II, s/n, Torre, 58040-440, João Pessoa, PB, Brazil; 6 Programa de Pós-Graduação em Zoologia, Centro de Ciências Exatas e da Natureza Universidade Federal da Paraíba, João Pessoa, PB, Brazil; 7 Coordenadoria de ordenamento pesqueiro/ Diretoria de fauna e recursos pesqueiros/Coordenação geral de gestão de recursos pesqueiros. SCEN, AV. L4 Norte, Edifício sede do IBAMA, Bloco B, subsolo, 70800-200, Brasília, DF. Caixa postal 09870, Brazil

## Abstract

From a conservationist perspective, seahorses are threatened fishes. Concomitantly, from a socioeconomic perspective, they represent a source of income to many fishing communities in developing countries. An integration between these two views requires, among other things, the recognition that seahorse fishers have knowledge and abilities that can assist the implementation of conservation strategies and of management plans for seahorses and their habitats. This paper documents the knowledge held by Brazilian fishers on the biology and ecology of the longsnout seahorse *Hippocampus reidi*. Its aims were to explore collaborative approaches to seahorse conservation and management in Brazil; to assess fishers' perception of seahorse biology and ecology, in the context evaluating potential management options; to increase fishers' involvement with seahorse conservation in Brazil. Data were obtained through questionnaires and interviews made during field surveys conducted in fishing villages located in the States of Piauí, Ceará, Paraíba, Maranhão, Pernambuco and Pará. We consider the following aspects as positive for the conservation of seahorses and their habitats in Brazil: fishers were willing to dialogue with researchers; although captures and/or trade of brooding seahorses occurred, most interviewees recognized the importance of reproduction to the maintenance of seahorses in the wild (and therefore of their source of income), and expressed concern over population declines; fishers associated the presence of a ventral pouch with reproduction in seahorses (regardless of them knowing which sex bears the pouch), and this may facilitate the construction of collaborative management options designed to eliminate captures of brooding specimens; fishers recognized microhabitats of importance to the maintenance of seahorse wild populations; fishers who kept seahorses in captivity tended to recognize the condtions as poor, and as being a cause of seahorse mortality.

## Introduction

Over a decade ago, Ruddle [1] pointed out the great potential value of local knowledge as an information base for local management of marine environments and resources, especially in the tropics, where conventionally-used data were usually scarse to non-existent. A number of subsequent studies have documented and recognized the value of local knowledge to conservation and management of fisheries [2-12].

A pragmatic view of the relevance of fishers knowledge to fisheries management has been expressed by Ames [8]: "fishermen and their subjective, anecdotal descriptions have a pivotal role to play in the development and function of sustainable fisheries (.......) fishermen are, in fact, the only available source of local, historical, place-based information". Nevertheless, lack of sound management practices have led to the collapse of particular types of fisheries in some parts of the world, and, as pointed out by Meewig *et al*. [13], interest in participatory approaches in resource management in part reflects the failure of top-down, centralized approaches to manage natural resources.

Definitions of artisanal, subsistence fisheries have traditionally focused on the capture and trade of food fish. However, a growing number of examples of fish species being traded worldwide for purposes other than alimentary (e.g., as pets, remedies, souvenirs) has revealed the existence of an international and multi-faceted commerce, supported by a diffuse (and generally poorly quantified) harvesting of a number of species.

Those forms of exploitation have received little attention when compared with the trade of animals for alimentary purposes [14]. In the marine realm, the scarcity of data has rendered the identification of key elements for conservation and management, and the assessment of impacts difficult.

Seahorses (*Hippocampus *spp.) are among the few non-food marine fishes whose trade has been documented, initially in Asia [15], where the demand for those fishes was primarily for use in the Traditional Chinese Medicine (TCM) and its derivatives.

Brazil has been involved in the dried seahorse trade, and has been a major exporter of live seahorses at least since 1999. However, only recently the magnitude and impacts of the seahorse fishery in the country began to be assessed and translated into regulatory measures [16]. The seahorse fishery involves many fishing communities in Brazil, to whom seahorses represent an important source of income, particularly in the Northeast of the country.

This paper represents the first attempt to use an ethnoecological approach to examine issues relevant to seahorse conservation and management in Brazil. Its aims were to explore collaborative approaches to seahorse conservation and management in Brazil; to assess fishers' perception on seahorse biology and ecology, in the context evaluating potential management options; to increase fishers' involvement with seahorse conservation in Brazil.

## Background

Seahorses (genus *Hippocampus*) are traded worldwide for use in traditional medicines, as aquarium pets and as curios. Initial surveys of the seahorse trade conducted in the nineties showed that the market for seahorses was economically important, threatened wild seahorse populations, and involved 32 countries [15]. Recent surveys have shown that the number of countries known to be involved in the trade has risen to at least 77 [17]. Additionally, seahorses' coastal habitats, such as reefs and mangroves, are among the most threatened in the world.

The combination of those two factors has resulted in the listing of 33 seahorse species on the IUCN Red List [18], and in the inclusion of the entire genus *Hippocampus *in the Appendix II of the Convention on International Trade in Endangered Species of Wild Fauna and Flora (CITES). Besides requiring that source countries demonstrate that exports are non-detrimental to the long-term persistence of wild seahorse populations, the listing highlighted the need for monitoring seahorse wild populations, so that the international seahorse trade can be effectively managed.

*Hippocampus reidi*, commonly known as the longsnout seahorse, is one of the most sought after seahorse species in the aquarium trade. The species figures in the IUCN Red List as Data Deficient [18].

The recognition that seahorses constitute an important source of income, and the need to ensure that their trade is non-detrimental to the long-term persistence of wild populations create a need to supplement the existing regulatory measures with other initiatives, such as cooperative resource management.

In recent years, researchers have emphasized the importance of the knowledge produced and orally transmitted by traditional fishermen and the potential role of traditional fishing and related environmental knowledge can play for the development and implementation of fisheries management [1,10,19-21,4-8]. With regards to seahorses, Pajaro *et al.*[2] and Meewig et al.[13] have highlighted the importance of fishers' knowledge to the conservation of those fishes in the Philippines.

Lessons learned through examination of the multi-faceted fishery and trade of seahorses can perhaps be used to increase our understanding of interesting questions encompassing social, economic, environmental and cultural aspects of other artisanal fisheries.

## Materials and methods

**Figure 1:**
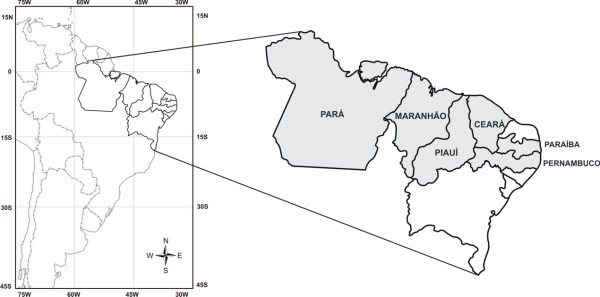
Map showing the surveyed States.

The study was based on field surveys conducted between February/2002 and October/2005 in fishing villages at the Northeastern Brazilian States of Piauí, Ceará, Paraíba, Maranhão and Pernambuco, and at the Northern State of Pará (Figure 1, Table 1), and focused on the species *Hippocampus reidi *Ginsburg, 1933.

**Table 1:** Localities where fishers were interviewed in Brazil.

**REGION / STATE / MUNICIPALITY**	**COORDINATES**	**LOCALITIES WHERE INTERVIEWS WERE MADE**
**NORTHEASTERN**		
**Ceará**		
Acaraú	02°53'08" S 40°26'57" W	Rio Acaraú, Arpoeiras
Aquiraz	03°54'05" S 38°23'28" W	Prainha, Iguape, Batoque
Beberibe	04°10'47" S 38°07'50" W	Parajuru, Rio Choro, Uruau, Sucatinga
Camocim	02°54'08" S 40°50'28" W	Rio Coreaú, Tatajuba
Cascavel	04°07'59" S 38°14'31" W	Rio Mal Cozinhado, Caponga, Balbino, Águas Belas
Caucaia	03°44'10" S 38°39'11" W	Rio São Gonçalo, Barra do Cauípe
Cruz	02°55'04" S 40°10'18" W	Preá
Eusébio	03°53'24" S 38°27'02" W	Rio Pacoti
Fortim	04°27'07" S 37°47'50" W	Rio Jaguaribe, Rio Piranji
Icapui	04°23'15" S 37°21'48" W	Manibu, Requenguela, Retiro Grande, Redonda, Peixe Gordo, Braço de mar da Barra Grande
Itarema	02°55'13" S 39°54'54" W	Braço de mar de Porto dos Barcos
Jijoca	02°53'42" S 40°26'57" W	Rio Guriu
Paracuru	03°24'36" S 39°01'50" W	Rio Curu
São Gonçalo do Amarante	03°36'26" S 38°58'06" W	Pecém, Taíba
Trairi	03°16'40" S 39°16'08" W	Rio Trairi, Rio Mundau, Guajiru, Flexeiras, Mundau
**Maranhão**		
Raposa	02°25'23" S 44°06'12" W	Raposa
**Paraíba**		
Marcação	06°48'11" S 35°04'50" W	Tramataia
Rio Tinto	06°48'11" S 35°04'50" W	Barra de Mamanguape
**Pernambuco**		
Goiana	07°33'38" S 35°00'09" W	Estuário de Itapessoca
**Piauí**		
Cajueiro da Praia	02°55'40" S 41°20'10" W	Barra Grande, Cajueiro de Cima
**NORTH**		
**Pará**		
Bragança	01°07'30" S 46°37'30" W	Rio Caeté, Rio Maguari, Praia de Ajuruteua
Augusto Corrêa	01°07'30" S 46°37'30" W	Rio Urumajó

Pilot surveys were conducted to delimit the areas where a seahorse fishery existed along the Brazilian coast. During that period (January–July/2002), observations of the application of ecological knowledge, and acquisition of baseline information on fishing communities were done in praxis.

Ethnoecological data were gathered through semi-structured questionnaires and semi-directive interviews, with some questions left open-ended [22-24]. Interviews were conducted on a one-to-one basis. Based on information provided by community leaders, we initially sought out intentional seahorse fishers, who exclusively collect or have collected seahorses over a period of time. A total of 36 intentional seahorse fishers was found. Subsequently, we interviewed fishers who occasionally come in contact with seahorses, either through their own fishing gear while harvesting for other resources, or as bycatch from the commercial shrimp, fish and lobster nets. Additional interviewees were chosen by using the snowball technique, based on information initially provided by the intentional fishers [25].

Throughout the study, 47 localities (22 municipalities) were visited, in which 181 fishers were interviewed (42 in Ceará State, 29 in Pernambuco, 29 in Maranhão, 19 in Paraíba, 32 in Piauí and 30 in Pará). Questionnaires and interviews encompassed questions on seahorse ethnotaxonomy, behavior, feeding ecology, reproduction and habitats. No information provided by repondents was excluded from the analysis, following Marques [26]. Interviewees sometimes provided more than one answer to the same question (i.e., seahorses inhabit estuaries and reefs), therefore in some cases the sum of sample sizes provided for a given answer may be higher than the total number of people interviewed.

### Surveyed communities

Our surveys focused on the North and Northeastern regions of Brazil, and encompassed six States (Figure 1). In four of the States seahorses were or are targetted by local fishers, while in the remaining States there was no fisheries directed to seahorses.

Pará State, N Brazil – Two localities were surveyed: Bragança and Augusto Corrêa, both portuary cities. Interviewees at the two localities did not target seahorses in their fisheries, nevertheless, seahorses were caught as bycatch in commercial shrimp, food-fish or lobster nets. Interviewed fishers came in contact with seahorses either through the incidental captures, or by direct observation in the wild while fishing for food. Seahorses obtained as bycatch in the two localities generally enter the dried trade, and occasionally were taken home by fishers to be used as medicine. Fishers' age ranged from 23 to 66 years. With regards to schooling, 43.3% (n = 13) of the fishers interviewed were illiterate, 6.7% (n = 2) attended school for eight years (completing what is known in Brazil as "ensino fundamental"), while 50% (n = 19) attended school for less than eight years.

Maranhão State, NE Brazil – The surveyed locality (Raposa municipality) encompasses the largest and most important fishing community in the State of Maranhão [27]. Seahorses were not targetted by the local fishery, nevertheless they were caught as bycatch in commercial shrimp, food-fish or lobster nets. Interviewed fishers came in contact with seahorses either through the incidental captures, or by direct observation in the wild while fishing for food. Seahorses obtained as bycatch generally enter the dried trade, and occasionally are taken home by fishers to be used as medicine.

Fishers' age ranged from 24 to 67 years. With regards to schooling, 31% (n = 9) of the fishers interviewed were illiterate, 6.9% (n = 2) attended school for eight years (completing what is known in Brazil as "ensino fundamental"), 58.6% (n = 17) attended school for less than eight years and 3.4% (n = 1) attended the three years of high school (completing what is known in Brazil as "ensino médio").

Piauí State, NE Brazil – Two localities were surveyed: Cajueiro da Praia and Barra Grande, both within the limits of an Environmental Protected Area. The main economic activities in that area are commercial fisheries and shrimp farms adjacent to the Timonha-Ubatuba estuarine system. Seahorses were targetted in the area, being sold as ornamental fish and/or dried; occasionally seahorses were taken home by fishers to be used as medicine. Interviewed fishers came in contact with seahorses through the seahorse fishery, local incidental captures, or by direct observation in the wild while fishing for food. Seahorses obtained as bycatch generally enter the dried trade, and occasionally are taken home by fishers to be used as medicine.

Fishers' age ranged from 19 to 76 years. With regards to schooling, 71.9% (n = 23) of the fishers interviewed were illiterate, and, 28.1% (n = 9) attended school for less than eight years.

Ceará State, NE Brazil – At Ceará 15 coastal municipalities were surveyed (Table 1). Fisheries conducted in Ceará's coastal areas are of great social importance [28]. Interviewed fishers came in contact with seahorses through the seahorse fishery, incidental captures (local or external to the community), or by direct observation in the wild while fishing for food.

With regards to schooling, 72.7% of the fishers interviewed atended school for less than eight years, 18.2% were illiterate and 9.1% attended school for 11 years (completing what is known in Brazil as "2° grau").

Paraíba State – The surveyed locality (Mamanguape Estuary, 06° 43' e 06°51' S e 35°07' e 34° 54' W), is part of an Environmental Protected Area. Interviewees belong to two communities located along opposite margins of that estuary, and have been traditionally involved with artisanal fisheries. Seahorses have been amply harvested in that area (the activity peaked from 1999 to 2003) and were mostly sold as ornamental fishes. Occasionally seahorses were taken home by fishers to be used as medicine. Interviewees came in contact with seahorses through the seahorse fishery, incidental captures, or by direct observation in the wild while fishing for food.

Age of interviewed fishers ranged from 30 to 65 years. With regards to schooling, 47.4% (n = 9) of the fishers interviewed were illiterate, 10.5% (n = 8) attended school for eight years (completing what is known in Brazil as "ensino fundamental"), 42.1% (n = 8) attended school for less than eight years.

Pernambuco State – At the surveyed locality (Itapessoca estuary, 07° 37', 07°41' S and 34° 50', 34° 55' W), all fishers interviewed were male, their age ranging from 16 to 69 years old. Artisanal fisheries constitute a main source of income in that area. Seahorses have been traditionally harvested at Itapessoca and sold as ornamental fishes (for at least 10 years); occasionally seahorses were taken home by fishers to be used as medicine. With regards to schooling, 10% (n = 3) of them were illiterate, 3% (n = 1) attended the four years of elementary school, 67% (n = 19) attended elementary for less than four years; 10% (n = 3) attended the three years of high school, while 10% (n = 3) attended high school for less than three years.

## Results and Discussion

Answers provided by fishers are summarized in Tables 2 and 3. Interviewed fishers said that they acquired their knowledge on the biology and ecology of seahorses by direct contact with the seahorse fishery (n = 36, 20%), through handling specimens caught as bycatch (n = 59, 32.5%), or by in direct contact with specimens the wild, while harvesting for other resources, such as molluscs, crustaceans or food-fish (n = 86, 47,5%).

**Table 2:** Summary of information provided by fishers in the surveyed communities in Brazil.

	**Regions / Brazilian States**					
	**NORTHEASTERN**					**NORTH**

	Ceará (n = 42)	Maranhão (n = 29)	Paraíba (n = 19)	Pernambuco (n = 29)	Piauí (n = 32)	Pará (n = 30)

**Intentional**	12		02	12	10	
**Occasional**	30	29	17	17	22	30
Age groups of fishers	10 a 20 (25%)	20 a 30 (10.3%)	30 a 40 (26.3%)	10 a 20 (10.3%)	10 a 20 (3.1%)	20 a 30 (23.3%)
	20 a 30 (50%)	30 a 40 (17.2%)	40 a 50 (26.3%)	20 a 30 (10.3%)	20 a 30 (15.6%)	30 a 40 (30%)
	30 a 40 (25%)	40 a 50 (41.4%)	50 a 60 (26.3%)	30 a 40 (31 %)	30 a 40 (15.6%)	40 a 50 (33.3%)
		50 a 60 (27.6%)	60 a 70 (21.1%)	40 a 50 (20.7%)	40 a 50 (25%)	50 a 60 (6.7%)
		60 a 70 (3.5%)		50 a 60 (20.7%)	50 a 60 (18.8%)	60 a 70 (6.7%)
				60 a 70 (7%)	60 a 70 (12.5%)	
					70 a 80 (9.4%)	
**Ethnoclassification**	Colour only (n = 20), colour and skin appendages (n = 12), no answers (n = 10)	Colour only	Colour only	Colour only (n = 10), colour and skin appendages (n = 19)	Colour only (n = 30), colour and skin appendages (n = 2)	Colour only
**Population decline**	Yes (n = 8), no (n = 10), no answers (n = 24)	Yes (n = 15), no (n = 14)	Yes (n = 19)	Yes (n = 25), no (n = 4)	Yes (n = 13), no (n = 19)	Yes (n = 8), no (n = 22)
**Cited habitats**	Only estuarine areas (camboas) (n = 26), only sea (n = 13), estuarine areas (camboas) and sea (n = 3)	Only estuarine areas (camboas) (n = 24), only sea (n = 1), estuarine areas (camboas) and sea (n = 4)	Only estuarine areas (camboas) (n = 3), estuarine areas (camboas) and sea (n = 16)	Only estuarine areas (camboas) (n = 29)	Only estuarine areas (camboas) (n = 30), estuarine areas (camboas) and sea (n = 2)	Only estuarine areas (camboas) (n = 12), only sea (n = 10), estuarine areas (camboas) and sea (n = 8)
**Sazonal migration**	Yes (n = 19), no (n = 2), no answers (n = 21)	No (n = 29)	Yes (n = 19)	Yes (n = 15), no (n = 1), no answers (n = 13)	No (n = 32)	No (n = 30)
**Cited preys**	No answers (n = 31), "dirt in the water" (n = 3), fish larvae (n = 1), shrimp larvae (n = 3), crabs (n = 2), algae (n = 2), "lodo" (n = 1)	No answers (n = 14), "dirt in the water" (n = 5), algae (n = 1), "lodo" (n = 11)	No answers (n = 4), fish and shrimp larvae (n = 6), algae (n = 4), "lodo" (n = 5)	No answers (n = 12), adult shrimp (n = 3), shrimp larvae (n = 11), algae (n = 1), mud and "lodo" (n = 2)	No answers (n = 7), "dirt in the water" (n = 5), shrimp larvae (n = 1), algae (n = 4), mud and "lodo" (n = 16)	No answers (n = 6), "dirt in the water" (n = 4), fish larvae (n = 1), shrimp larvae (n = 1), algae (n = 7), mud and "lodo" (n = 19)
**Cited Predators**	No answers (n = 33), "baiacu" (n = 4), "moréias" (n = 3), "ciobas" (n = 1), "cavalas" (n = 1), "beijupirás" (n = 1), other fishes (n = 1)	No answers (21), none (n = 1), large fish ("mero") (n = 1), other fishes (n = 3), crabs (n = 3)	None (all answers)	No answers (n = 21), none (n = 6), crabs (n = 1), large fish ("mero") (n = 1)	No answers (n = 20), none (n = 3), large fish ("mero") (n = 1), "baiacu" (n = 4), crabs (n = 2), "pacamão" (n = 2), "dourado velho" (n = 1), "bagre" (n = 3), "carapitanga" (n = 1), "ariacó" ("cioba") (n = 1)	No answers (n = 11), none (n = 1), "pescada amarela" (n = 3), "garoupa" (n = 4), "gorijuba" (n = 3), "cação" (n = 6), "camurupim" (n = 1), "piraúna" (n = 1), "pargo" (n = 2), "sirigado" (n = 1), large fish ("mero") (n = 1), "baiacu" (n = 2), "bagre" (n = 1), "ariacó" ("cioba") (n = 3), other fishes (n = 5), crabs (n = 1)
**Feeding behavior**	Not mentioned	Not mentioned	One respondent	Not mentioned	Not mentioned	Not mentioned
**Social structure (adults)**	Solitary (generally) (n = 12), groups of 2 or 3 individuals (n = 5), solitary and groups of 2 or 3 individuals (n = 1), no answers (n = 24)	Solitary (n = 14), groups of 2 a 5 individuals (n = 11), solitary and groups of 2 or 3 individuals (n = 4)	Not mentioned	Solitary (n = 21), groups of 2 a 3 individuals (n = 6), solitary and groups (n = 1), no answers (n = 1)	Solitary (n = 12), groups of 2 a 3 individuals (n = 16), solitary and groups of 2 a 3 individuals (n = 4)	Solitary (n = 24), groups of 2 a 3 individuals (n = 5), solitary and groups of 2 or 3 individuals (n = 1)
**Pregnancy**	No answers (n = 28), male (n = 11), female (n = 3)	Female (n = 29)	Male (n = 2), female (n = 17)	No answers (n = 6), male (n = 11), female (n = 12)	Male (n = 3), female (n = 29)	Male (n = 0), female (n = 30)
**Courtship behavior**	Not mentioned	Not mentioned	Mentioned by one respondents	Not mentioned	Mentioned by three respondents	Not mentioned
**Reproductive period**	No answers (n = 31), winter (n = 7), summer (n = 1), throughout the year (n = 3)	No answers (n = 21), winter (n = 5), summer (n = 3)	No answers (n = 2), summer (n = 16), throghout the year (n = 1)	No answers (n = 22), winter (n = 5), summer (n = 1), throghout the year (n = 1)	No answers (n = 23), winter (n = 6), summer (n = 1), throughout the year (n = 2)	No answers (n = 23), winter (n = 3), summer (n = 3), throughout the year (n = 1)

**Table 3:** Summary of information provided by fishers (intentional and occasional) in the surveyed communities in Brazil.

	**Brazilian States**							
	**Paraiba (n = 19)**		**Pernambuco (n = 29)**		**Ceara (n = 42)**		**Piauí (n = 32)**	

	Intentional fishers (n= 2)	Occasional fishers (n = 17)	Intentional fishers (n= 12)	Occasional fishers (n = 17)	Intentional fishers (n= 12)	Occasional fishers (n = 30)	Intentional fishers (n = 10)	Occasional fishers (n = 22)

Ethnoclassification	Colour (n = 2)	Colour (n = 17)	Colour (n = 3), colour and skin appendages (n = 9)	Colour (n = 7), colour and skin appendages (n = 10)	Colour (n = 2), colour and skin appendages (n = 10)	Colour (n = 19), colour and skin appendages (n = 2), No answers (n = 9)	Colour (n = 8), colour and skin appendages (n = 2)	Colour (n = 22)
Population decline	Yes (n = 2)	Yes (n = 17)	Yes (n = 9), no (n = 3)	Yes (n = 16), no (n = 1)	Yes (n = 5), no (n = 6) no answers (n = 1)	Yes (n = 3), no (n = 4) no answers (n = 23)	Yes (n = 8), no (n = 2)	Yes (n = 5), no (n = 17)
Habitats	Estuarine areas only (camboas) (n = 2)	Estuarine areas (camboas) and sea (n = 17)	Estuarine areas only (camboas) (n = 12)	Estuarine areas only (camboas) (n = 17)	Estuarine areas only (camboas) (n = 12)	Estuarine areas only (camboas) (n = 12), sea only (n = 12), estuarine areas (camboas) and sea (n = 6)	Estuarine areas only (camboas) (n = 9), estuarine areas (camboas) and sea (n = 1)	Estuarine areas only (camboas) (n = 21), estuarine areas (camboas) and sea (n = 1)
Seasonal migration	Yes (n = 2)	Yes (n = 17)	Yes (n = 9), no answers (n = 3)	Yes (n = 5), no (n = 1), no answers (n = 11)	Yes (n = 9), no (n = 1), no answers (n = 2)	Yes (n = 10), no (n = 1), no answers (n = 19)	No (n = 10)	No (n = 22)
Quoted prey types	Shrimp larvae (n = 2)	Shrimp and larvae (n = 4), algae (n = 4), "lodo" (n = 5), no answers (n = 4)	Adult shrimp (n = 2), shrimp larvae (n = 7), algae (n = 1), no answers (n = 2)	Adult shrimp (n = 1), shrimp larvae (n = 4), mud and "lodo" (n = 2), no answers (n = 10)	Fish and algae (n = 1), shrimp larvae (n = 3), crabs (n = 2), "dirt in the water" (n = 3), "lodo" (n = 1), no answers (n = 2)	"Dirt in the water" (n = 1), no answers (n = 29)	Egg of fish (1), algae (n = 1), "dirt in the water" (n = 1), mud and "lodo" (n = 6), no answers (n = 1)	"Dirt in the water" (n = 4), lama and "lodo" (n = 10), shrymp larvae (1), algae (n = 3), no answers (6)
Quoted predators	None (all answers)	None (all answers)	None (n = 5), large fish ("mero") (n = 1), no answers (n = 6)	None (n = 1), crabs (n = 1), no answers (n = 15)	"Baiacu" (n = 4), "moréias" (n = 3), no answers (n = 5)	Other fishes (n = 1); "cioba", "cavala" e "beijupirá" (n = 1), no answers (n = 28)	"Baiacu" (n = 1), "dourado velho" (n = 1), "bagre" (n = 1), "carapitanga" (n = 1), "siri" (n = 2), no answers (n = 7)	"Baiacu" (n = 3), "pacamão" (n = 2), "bagre" (n = 2), "cioba" (n = 1), no answers (n = 16)
Feeding behavior	One respondent	Not mentioned	Not mentioned	Not mentioned	Not mentioned	Not mentioned	Not mentioned	Not mentioned
Social structure (adults)	Not mentioned	Not mentioned	Solitary (generally) (n = 7), groups of 2 or 3 individuals (n = 5)	Solitary (generally) (n = 14), groups of 2 or 3 individuals (n = 1), solitary and groups of 2 or 3 individuals (n = 1), no answers (n = 1)	Solitary (generally) (n = 4), groups of 2 or 3 individuals (n = 5), solitary or in groups of 2 or 3 individuals (n = 1), no answers (n = 2)	Solitary (generally) (n = 8), no answers (n = 22)	Solitary (generally) (n = 3), groups of 2 or 3 individuals (n = 7)	Solitary (generally) (n = 9), groups of 2 or 3 individuals (n = 9), solitary or in groups of 2 or 3 individuals (n = 4)
Presence of brooding pouch	Female (n = 2)	Male (n = 1), female (n = 16)	Male (n = 6), female (n = 4), no answers (n = 2)	Male (n = 5), female (n = 8), no answers (n = 4)	Male (n = 10), female (n = 2)	Male (n = 1), female (n = 1), no answers (n = 28)	Male (n = 3), female (n = 7)	Female (n = 22)
Courtship behavior	One respondent	Not mentioned	Not mentioned	Not mentioned	Not mentioned	Not mentioned	Three respondents	Not mentioned
Reproductive period	Summer (n = 2)	Summer (n = 14), throughout the year (n = 1), no answers (n = 2)	Winter (n = 1), throughout the year (n = 1), no answers (n = 10)	Summer (n = 1), Winter (n = 4), no answers (n = 12)	Summer (n = 1), Winter (n = 6), throughout the year (n = 3), no answers (n = 2)	Winter (n = 1), no answers (n = 29)	Summer (n = 1), Winter (n = 1), throughout the year (n = 2), no answers (n = 6)	Winter (n = 5), no answers (n = 17)

### Ethnotaxonomy

The first frame of reference for gathering and organizing traditional environmental knowledge is taxonomic [29]. The classification of plants and animals by traditional societies is viewed as a reflex of cognitive and intellectual principles to understand the world, being mainly moved by "interest" [30]. In the present study most interviewees (N = 171, 94.5%) said that they recognized different types of seahorses; ten fishers (5.5%) from Ceará State did not answer the question on ethnoclassification. All interviewed fishers (n = 181) used colour to differentiate morphotypes; among them, 33 (19.3%) also used the presence of skin appendages to differentiate morphotypes. Fishers who targetted seahorses for the live trade recognized colourful seahorses as more valuable than black seahorses; 72.2% of them (n = 26), said that seahorse wild populations had declined over the years, affecting their capacity to choose seahorses by colour.

The number of colour morphs recognized by fishers ranged from five to 10, and closely corresponded to the colour patterns recorded to *H. reidi *by [31]. Generally, categories were consistently employed by fishers, irrespective of fishers being intentional or occasional, of differences in age or circumstances of use. At Pará nine colour morphs were recognized (black, grey, yellow, brown, orange, red, green, white and burgundy), while at Maranhão fishers recognized seven colour patterns (black, grey, yellow, yellow with darker dots, red, white and white with darker dots). Fishers from Piauí mentioned eight colour morphs (black, black with white dots, yellow, brown, brown with lighter dots, red, greenish and whitish), while fishers from Ceará recognized five colour morphs (black, yellow, red, black and white, and red and yellow); fishers from Paraíba mentioned five colour morphs (black, yellow, yellow with black marks, red, and green), while fishers from Pernambuco recognized 10: black, grey, yellow, brown, red, red with black dots, white, orange and white, orange and green and burgundy. Seahorses are cryptic species which use camouflage as a defense mechanism Their colour patterns can be highly variable intraspecifically [32].

**Figure 2:**
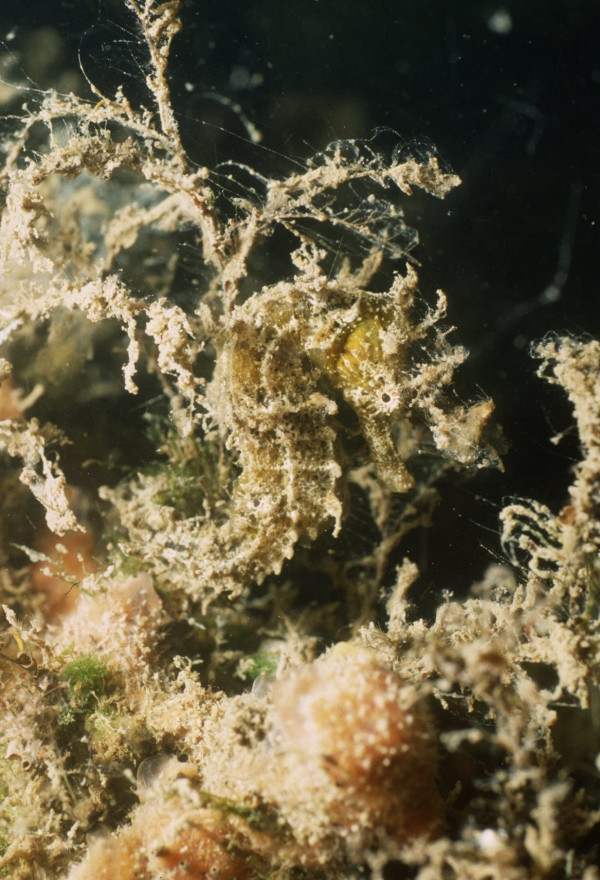
Camouflaged *Hippocampus reidi*, showing skin appendages. Photo: Bertran M. Feitoza.

Knowledge about the presence of skin appendages was more pronounced among intentional fishers. Skin appendages are known to occur in a number of seahorses species, and can also vary intraspecifically. Their presence adds up to seahorses' ability to blend in with their surroundings (see Figure 2), therefore it would be expected that fishers who targetted seahorses would be more knowledgeable about that characteristic than occasional fishers – a reflex of their greater ability to find seahorse specimens in the wild.

A field study conducted at Rio Grande do Norte, NE Brazil [33], found that most specimens with skin appendages were juveniles, a view only expressed by one fisher from Ceará. One fisher from Pernambuco associated skin appendages with older seahorses; at Ceará, seven fishers indicated that skin appendages were found in black specimens, an information that generally concides with the results of underwater surveys conducted in that State, where most specimens with skin appendages were either black or brown. Fishers from Pernambuco mentioned that skin appendages were very common, and that "nearly all seahorses have them". That information agrees with data obtained through underwater surveys, and should be further investigated from a taxonomic viewpoint.

### Habitat

Seahorses are cryptic species, which are known to have a patchy distribution, site fidelity, small home ranges and low mobility [34]. Therefore, we antecipated that intentional fishers have precise knowledge about seahorses' habitat use.

All fishers interviewed mentioned that seahorses were sedentary animals which used their tail to grab holdfasts (e.g., roots or leaves of mangrove trees, algae, corals and rocks), or that are seen floating or leaning their bodies against the muddy substrate. A comparison of fishers' perception with data available in the scientific literature [35-38] reveals that the two sources of information were generally consistent with each other.

**Figure 3:**
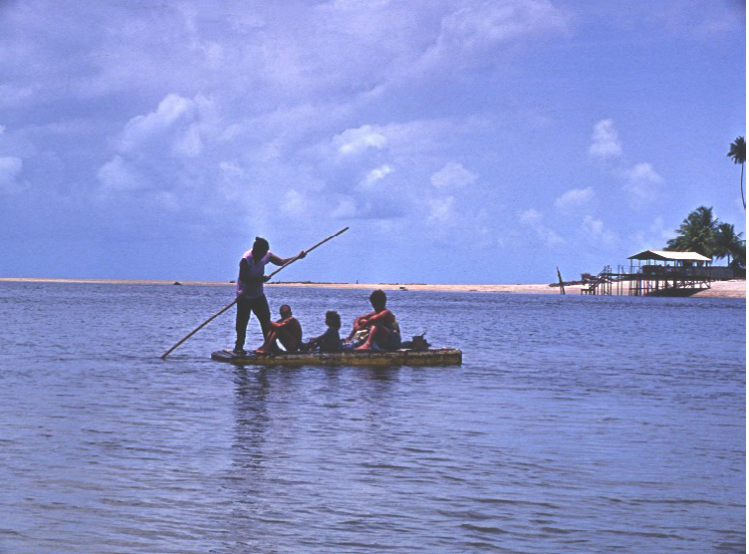
Seahorse fishers, Pernambuco State. Photo: Bertran M. Feitoza.

Most respondents said that seahorses exclusively inhabit shallow estuarine areas (Figure 3), particularly along their margins or in places where currents are not strong – locally known as "camboas" (n = 124, 68.5%). 13.2% of the fishers (n = 24) said that seahorses lived exclusively in the sea, while 33 (18.3%) mentioned that seahorses inhabit both estuaries and the sea. Among the fishers who mentioned that seahorses live in the sea, 14 from Pará, five from Maranhão, 13 from Ceará and 12 from Paraíba mentioned that those fishes inhabit deep waters (up to 80 m), being incidentally captured in nets in areas where algae are found. One fisher from Pará and 10 from Ceará added that seahorses sometimes are found in traps designed to capture other fish species or lobster, locally known as "manzuá"; three fishers (two from Pernambuco and one from Pará) mentioned that seahorses sometimes use the screens of fish corrals as anchor points. The incidental capture of seahorses in shrimp and occasionally in lobster nets has been reported in Brazil [16], however, neither accidental captures in traps nor the occurrence of seahorses in fish corrals had been previously reported in the country.

On the other hand, four fishers from Paraíba, four from Pará, two from Piauí and three from Ceará quoted shallow areas (including the intertidal zone) as seahorses' habitat. Some fishers (16 from Ceará and one from Piauí) added that seahorses can also be be sighted from the boats, near the sea surface. Most habitats quoted by fishers coincide with the scientific literature [35-38], except for their occurrence in the intertidal zone.

*H. reidi *is known to occur in a relative wide range of depths (10 cm to 60 m) and salinities (up to 45‰) in Brazilian waters [16,36]. These variations were encompassed by the answers provided by fishers, and possibly reflect the types of habitats used by them as fishing areas (i.e., intentional fishers only harvesting specimens in estuaries, and occasional fishers using both the estuary and the sea as fishing grounds).

Locations of rare or endangered species are more likely to be identified by local resource users involved in such mapping exercises than by outside researchers doing site inventories [29]. The knowledge held by fishers on the distribution and characteristics of different microhabitats used by *H. reidi *can provide effective shortcuts for researchers investigating the local resource base, and be instrumental to the mapping of seahorses' geographic and spatial distribution. This is of particular relevance in a country with one of the longest coastlines in the world, and with limited resources for conducting surveying and population monitoring.

All respondents reported that seahorses cannot survive in freshwater. However, intentional fishers could provide more information on seasonal migrations. Intentional fishers who said that seahorses migrated (n= 20, 55.5%) provided three diferent answers: migrations occurred a) during the winter, when seahorses moved to the river's mouth or the sea "looking for higher salinity" b) during the summer or c) throughout the year (see Table 3). Vertical migration to deeper, more saline portions of the water column has been suggested to occur in *H. reidi *[31]; other seahorse species, such as *H.comes*, *H.erectus *and *H. whitei *are known to have made some seasonal migration in the winter [34].

The information provided by interviewees on *H. reidi'*s possible migratory patterns should be further investigated, as it has direct implications for the interpretation of local fisheries data, to the development of management options encompassing temporal closures, and to increase our understanding of *H. reidi'*s population dynamics. As pointed out by Johanes [29], animal migration pathways and aggregation sites known to local people will not always coincide with areas judged to be important based on common criteria for identifying sensitive areas such as aesthetic qualities or species diversity. However, in these areas the value of the resources which are known to local people is sometimes very great.

Interviewed fishers either walked to their fishing areas or used small boats, which they could manouver in shallow waters. Seahorses were generally hand-picked or caught with the aid of throw-nets dragged along the margins of the estuaries, which were recognized by fishers as areas where seahorses occur. Fishers' knowledge on *H. reidi'*s preferential habitats was used to maximize captures, while minimizing the time spent in the captures.

Water visibility and tides played an important role in the organization of the seahorse fishery. The influence of the tides on the activities carried out by fishers has been reported in the literature [6,39-41], and in the present study most (n = 176, 97.2%) of the interviewees mentioned that the tide directly influenced in the organization of their fishing activity.

### Feeding ecology

Seahorses are voracious carnivores, preying upon crustaceans, larval fishes and plankton. The few studies on their feeding ecology suggest that they may play a substantial role in structuring at least some benthic faunal communities [34].

Interviewees provided relevant information on *H. reidi*'s' diet, a still unstudied aspect of that species' ecology. The food items quoted by them generally agreed with the items reported for other species of the family Syngnathidae. Wilson and Vincent [42] showed that amphipods and copepods were the main food items consumed by *H. erectus*, and Kendrik and Hyndes [43] showed that small crustaceans dominated the diets of 12 syngnathid species. Two items quoted (fish eggs and larvae) had not been previoulsly recorded in the scientific literature.

Knowledge of how food was ingested by *H. reidi*, on the other hand, was virtually non-existent among interviewees. Only one respondent at Paraíba said that during feeding seahorses sucked prey into the "long thing" (meaning tubular snout), produced a snapping sound ("estralo"), and then swallowed the prey, a description that can be also found in Lourie *et al.*[32].

### Captivity care

All 36 intentional fishers provided information on captivity care. According to them, seahorses were kept in plastic or styrofoam containers, in glass aquaria equipped with an air pump (in most cases supplied by the buyer with whom the fisher has a non-written exclusivity "contract"), in PVC tanks or in containers ("gaiolas") left in the natural habitat; four intentional fishers from Piauí added that when the activity started, seahorses were kept in shrimp ponds of a local aquaculture farm owned by the main marine ornamental fish exporters from Ceará.

With regards to food items provided by seahorses, one fisher from Pernambuco said that seahorses were not fed in captivity; five reported that seahorses were fed in captivity, but did not specify which type of food they provided to the specimens; two fishers from Cajueiro da Praia mentioned that seahorses were fed with *Artemia *(supplied by the buyer), while eight did not know if seahorses were fed or not, because they handed the specimens they collected to other fishers who had tanks at home. *Artemia nauplii *has been widely used in seahorse aquaculture [42,44-46]. An *Artemia *only diet, however, is considered as nutritionally deficient [44,47]. Five fishers (four from Pernambuco and one from Paraíba) mentioned that seahorses were fed with "newly hatched" shrimp. One fisher from Pernambuco mentioned that seahorses were fed with "small fish". The other intentional fishers did not provide information on this topic.

According to two respondents from Piauí State, mortality rate in the tanks could reach 13%, inadequate feeding and poor sanitary conditions possibly playing a role in the mortality rate reported by the respondents. They also added that on one occasion all 700 seahorses they had kept for 15 days died, possibly due to the poor quality of the water in the tank.

**Figure 4:**
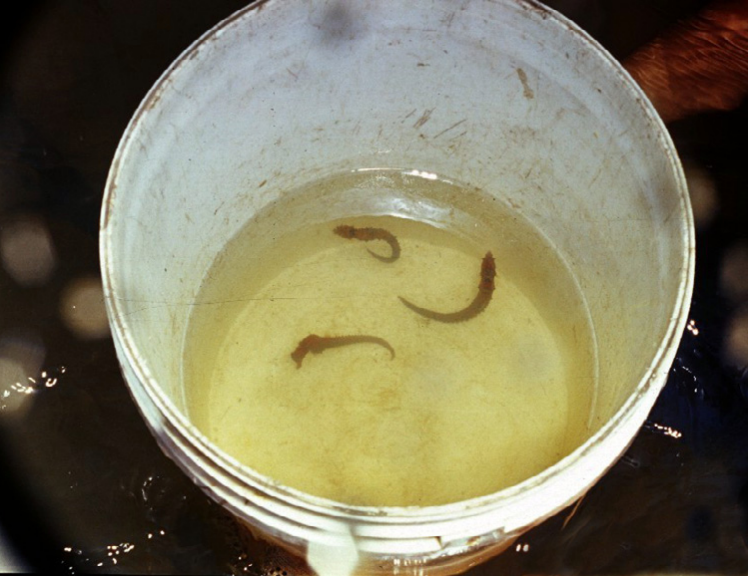
Type of container commonly used to keep the specimens of *Hippocampus reidi *harvested for the aquarium trade. Photo: Bertran M. Feitoza.

Fishers said that although they contacted buyers when specimens were available, in some cases they had to keep the seahorses for up to 15 days. Only 13.8 % of the intentional fishers (three from Pernambuco and two from Piauí) mentioned that they kept brooding seahorses until release of the offspring, and then released the young in the wild. At first glance, release of young seahorses in the estuary where the adults were captured may look like a good conservationist practice. However, unplanned release of organisms in the wild can cause a number of problems, particularly in cases where capitivity conditions are poor (see figure 4, as an example).

Maintenance of seahorses in their natural habitat reported by some interviewees may be more related to survival of the specimens (readily available food), and to limitations imposed by a few buyers with regards to purchase of brooding seahorses, rather than to conservationist reasons. Nevertheless, the initiative to keep the specimens in their natural habitat is positive, particularly given the present poor conditions and equipment available to fishers to maintain seahorse specimens in captivity, and should be further discussed with fishers.

### Predation

Most fishers (n = 106, 58.5%) did not provide information on predators of adult seahorses, and 16.6% (n = 30) of them said that the adult seahorse had no predators. The respondents who mentioned the existence of predators (n = 45, 24.8%) said that "seahorses were often found with cuts on the tail, body or belly, the wounds being inflicted by crabs" (*Callinectes *sp.), or mentioned that crabs and fishes fed on seahorses, the fishes being cobias (*Rachycentron canadum*), pufferfishes (*Sphoeroides *sp, *Colomesus psittacus*), morey eels (Muraenidae), mackerels (*Scomberomorus *sp.), groupers (*Epinephelus*), snappers (*Lutjanus *sp.), "gurijuba" (a type of catfish) "piraúna" (*Cephalopholis fulva*), cação (small shark), pacamão (*Amphichthys cryptocentrus*) and pescada amarela (Sciaenidae). Five fishers (two from Pará, one from Maranhão, and one from Piauí) said that they had found seahorse specimens in the stomach of food-fish. Zavala-Camin [48] recorded the presence of seahorse in the stomach of *C. hippuru*s, and two adult specimens of *H. reidi *have been found in the stomach of that same species (I. L. Rosa, unpublished data), thus corroborating the information provided by the fishers.

Crabs and seahorses share the same microhabitats in the surveyed estuaries, and therefore the wounds observed by fishers could be the result of agonistic interactions. However, although adult seahorses are recognized as having few predators [32], crabs and large pelagic fishes are considered as such in the literature [49]. Direct observations of seahorses with shortened tails also suggest that partial predation by crabs may be a threat to seahorses [50].

With regards to young seahorses, only three respondents could provide information on predation. According to them, soon after birth many young seahorses are rapidly eaten by other fish species, such as *bagres *(Ariidae), *carapitanga (Lutjanus *sp.*) *and *dourado velho (Coryphaena *sp.*)*. Those remarks agree with Lourie *et al.*[32], who mention that after birth seahorses receive no parental care, being very vulnerable to predation at that life stage.

Although cannibalism had not been previously recorded to *H. reidi *in the wild, two intentional fishers from Piauí said that young seahorses may be eaten by the adults: "the large seahorse eats the young ones. A young seahorse might be eaten if it stays in front of the adult". Interestingly, the two fishers observed cannibalism while seahorses were in captivity, and perhaps cannibalism occurred because the adult seahorses were underfed.

It is known that seahorses mostly rely on camouflage to avoid predators. A single mechanism of defense has been suggested in which, when threatened, seahorses react by bending their head and withdrawing their tail [32]. This agrees with the view expressed by fishers in this study, who said that seahorses were extremely "tame", not possessing any type of defense.

### Reproduction

Most respondents (n = 144) said that seahorses are born in the estuary; nine said that seahorses are born in the sea, and 28 did not answer the question. The dominant perception that seahorses are born in the estuary possibly reflects the fact the most interviewees use the estuary as fishing ground, where they encounter the young seahorses.

Only two fishers (from Piauí) could provide information on brood size, and that knowledge was acquired when keeping "pregnant" seahorses in tanks (and posteriorly releasing the offspring in the wild). According to the two fishers, the number of newborn young ranged from 800 to 1,000 per "pregnancy". The same respondents said that although "many seahorses are born, more than 100 at each contraction", after birth, "less than 1/4 survive" in the wild.

Seven respondents (three from Pará, two from Piauí and two from Pernambuco) said that the offspring looks like the adult specimens – "seahorses are born perfect, they are born complete". Two other respondents (from Pernambuco) mentioned that a young seahorse resembles a "mosquito larva". The information provided by fishers agrees with the literature. Seahorses are born as miniature adults, and during their initial phases mortality rates are high [32].

Egg-bearing *H. reidi *are known to occur in Brazilian estuaries (I. L. Rosa, unpublished data), and gives birth to approximately 1000 offspring at each "pregnancy" [51]. Teixeira and Musick [52] recorded 97 to 1552 eggs/embryos in *H. erectus*. Foster and Vincent [34] mentioned that, after brooding, male seahorses released from c. 5 to 2000 young, depending on species and adult size, and that newborn young measured from 2 to 20 mm in length. Fishers' limited knowledge on *H. reidi*'s initial phases evidentiated the need to develop educational activities in the surveyed communities to raise awareness with regards to seahorses' relatively low reproductive rates and high mortality during the initial phases, and the links between such life-history, fisheries and conservation.

### Sexual behavior

Some species of seahorse have elaborate courtship behavior, which includes daily greetings [53,54]. Only four of the intentional fishers (three from Piauí and one from Paraíba) knew about seahorses' courtship behavior. One described the behavior as "seahorses are always in the company of each other, sometimes males and females are together, I have seen them with their tails entangled". This description agrees with the observations made by Dias [31] who mentioned that males and females of *H. reidi *sometimes move as a pair, with their tails entangled. No occasional fisher could provide information on that topic.

It is known that *H. reidi*'s reproductive period lasts over eight months, and varies with temperature [51]. In Brazil, the species reproduces year-round (I. L. Rosa, unpublished data). Of the total number of interviewees, only 59 (22.6%) could provide information on seahorses' reproductive period. From these, 44.1%, (n = 26) mentioned that seahorses reproduced in the winter, while 42.4% (n = 25) said that seahorses reproduced during the summer. Eight fishers said that seahorses reproduced throughout the year. Among intentional fishers, 50% (n = 18) could provide information on seahorses' reproductive period.

### Sex differentiation

**Figure 5:**
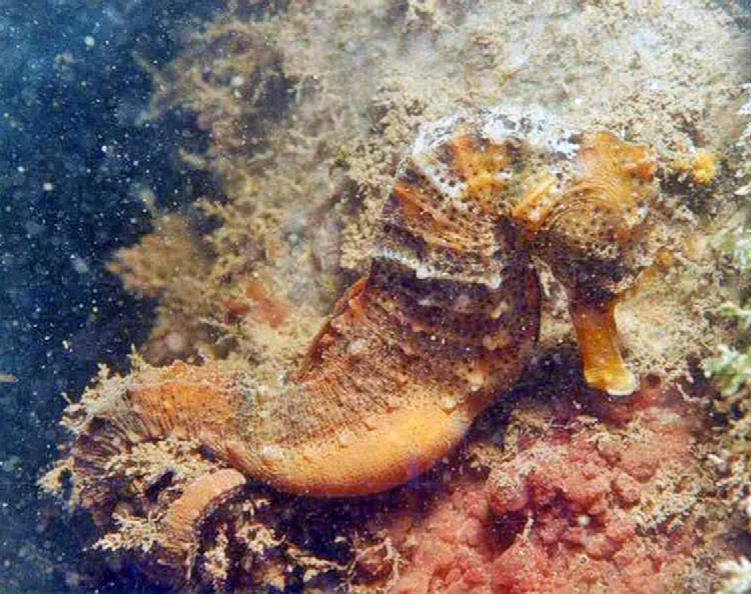
"Pregnant" male of *Hippocampus reidi *exhibiting ventral pouch. Photo: Bertran M. Feitoza.

Seahorses exhibit a unique reproductive system in which males have a ventral pouch (Figure 5) where the eggs are nourished [32,42,51]. Out of the 181 fishers interviewed, 147 (81,21%) said that they could differentiate males from females by the presence of a ventral pouch in the former. 120 (81.6%) associated the presence of the ventral pouch with the female seahorse – "females have the pouch, they are the ones that caries the embryos – the natural way". Among the intentional fishers (n = 36), 15 (41.6%) associate the presence of the ventral pouch with the female seahorse, 19 (52.7%) associated it with the male seahorse. Two fishers did not answer the question. Among occasional fishers (n = 145), 105 (72.4%) associated the presence of the pouch with the females, while only eight (5.5%) associated it with the male seahorse. A total of 32 occasional fishers (22.1%) did not provide information on the topic.

The prevailing view among interviewd fishers was that the female seahorse has the pouch (as expressed by some fishers, the "natural way"). Although that type of perception can sometimes be related to "machismo" [20], some fishers admitted that they were changing their opinion, because "a television program said that the male seahorses are the ones that have the pouch". At the Raposa locality (where seahorses were not targetted), all of the respondents said that they could differentiate a male seahorse from a female, only one respondent of them said that they learned how to recognize males and females from a TV show. At Ceará, out of the 11 fishers who said that the male seahorse has the pouch, nine said that they learned it from the buyers (previously they believed that the females had the pouch), the same being the case with the three fishers from Piauí who mentioned that the male seahorse had the pouch. The changing perception on the seahorse pouch (female *versus *male) agrees with what has been pointed out by Johannes [55]- that traditional knowledge may be biased due to cultural influences, consumers'market, fishing gear, individual preferences and ability to observe and memorize information. Two other aspects that may influence traditional knowledge are ease of observation and perceived importance of a problem [56,57].

Martin-Smith *et al.*[58] have shown that in the Philippines, seahorse fishers understand that the "pregnant" males are important to the sustainability of the wild populations, and relate population declines to habitat damage.

Although only a small percentage of the fishers interviewed could provide information on seahorses' reproductive period, all of them recognized the presence of the brooding pouch as an indicator of reproduction; additionally, all intentional fishers understood that brooding specimens were important to the sustainability of the wild populations (nevertheles, 50% (n = 18) of them said that they collected "pregnant" specimens). Intentional captures of "pregnant" seahorses have also been recorded in other parts of Brazil [16]. As an exporter country, Brazil has to comply to the listing of seahorses in appendix II of CITES, and demonstrate that captures of seahorses are non-detrimental to wild populations. Therefore, feasible management options addressing the capture of brooding seahorses should be sought after in the country.

### Social structure

With regards to social structure, 137 (75.7%) of the interviewees provided information on social structure, while 24.3% (n = 44) did not provide information. Among the fishers who provided information, 60.6% (n = 83) respondents said that adult seahorses are solitary, 31.4% (n = 43) said that seahorses formed groups, generally with two or three individuals, and a maximum of six individuals, and 8% (n = 11) said that seahorses could either be found solitary or in groups. That perception is corroborated by scientific studies conducted in Brazil [31,36], in which *H. reidi *was found solitary or in small groups of up to 4 individuals.

### Population declines

Despite the importance of seahorses for some of the interviewees, 48.6% of the respondents (n = 88) mentioned that seahorse populations had declined over the years.

Intentional seahorse fishers 66.6% (n = 24) said that populations have declined over the years, their present catch levels being much smaller than they used to be, and their capacity to choose seahorses by colour reduced. The following causes for the declines were mentioned by fishers: habitat degradation (aquaculture ponds, siltation, mangrove destruction, use of the natural ichthyocide known in the Amazon region as "timbó"), exploitation (too many seahorses captured for the live trade or bycatch).

In practical terms, the population declines mentioned by fishers may result in the development of a less selective fishery in terms of colour and size among fishers who harvest seahorses for the aquarium trade. Colourful seahorses attain higher monetary value than black ones, therefore a reduction in their availability could lead to the capture of a higher number of specimens to minimize monetary losses. This aspect, when coupled with the non-selectivity of the dried trade of seahorses captured as bycatch in Brazil, could further impact wild populations of seahorses in the country. Nevertheless, unlike many food fish species targetted by artisanal fishers, seahorses require almost no gear, are comparatively easier to catch, and have a comparatively high economic value. Recognition of population declines by fishers (which are also recognized by researchers and decision-makers) perhaps could be used as a point of consensus upon which dialogue can be initiated. Examples of potential uses for the information provided by fishers to the manangement of seahorses and their habitats in Brazil are shown in Table 4.

**Table 4:** Examples of how information provided by fishers can be used to manage seahorses and their habitats in Brazil

**Objective**	**Information provided by fishers**
Identify threats to seahorses populations Determine those populations targetted by fisheries, the incidental capture in fisheries, and other sources of mortality	Population declines through overharvesting and bycatch; habitat damage.
Identify and document useful practices for maintaining seahorses in captivity (live trade)	Keeping seahorses in the wild until selling the specimens
Identify economic incentives that threaten seahorse wild populations	Brooding seahorses are captured because they are accepted by buyers; No control of seahorses caught as bycatch in commercial nets; specimens enter the dried trade
Reduce to the greatest extent practicable the incidental capture and mortality of seahorses in nets through the development of spatial and seasonal closures	Fishers detain a broad knowledge of seahorses' habitats and main areas of occurrence.
Establish necessary measures to protect and conserve seahorse habitats, through the identification of areas of critical habitat.	Fishers detain a broad knowledge of seahorses' habitats and main areas of occurrence, and of possible migrations
Gather information on seahorse populations and their habitats Initiate and/or continue long-term monitoring of priority seahorse populations	Fishers' knowledge on seahorses' habitat use, colour patterns and skin filaments can be used to monitor seahorse populations, and to better delimit seahorse populations from a taxonomic viewpoint.

## Conclusion

All interviewed fishers considered seahorses important either for their economic value or for their value as a medicinal resource for the community. Fishers collectively demonstrated a broad knowledge of the ecology of *Hippocampus reidi*, and comparison of ethnoecological data with scientific publications showed them to be compatible in most cases. Limitations of the ethnoecological data set included a shortage of useful information on feeding behaviour and courtship behaviour in all surveyed localities.

Most of the ethnoecological information provided by fishers appeared to be acquired through personal experience; in the only case where information was aquired through means other than personal experience, fishers were open and specific about the other sources of information (television, dealers).

Similarly to what has been found by Donovan and Puri [59] in a study on non-timber forest products in Indonesian Borneo, intentional seahorse fishers' traditional knowledge was primarily associated with exploitation, and encompassed aspects relevant to the location and collection of seahorses (e.g., preferential habitats, colour patterns, companion species). In other words, they knew how, where and when to fish for seahorses.

With regards to the capture of seahorses for ornamental purposes, although interviewed fishers apparently understood the links between uncontrolled harvesting and the present availability of seahorses, they still harvested specimens they themselves perceive as important to the persistence of seahorses' wild populations. Guidelines, mainly through education, must be designed to reduce the impacts of local resource users, and, more important, strategies to empower the local communities must be developed.

The decline of the artisanal fisheries focused on food fish has opened new "exploitation niches" among fishing communities in Brazil, the marine ornamental trade being one of them. However, unlike other categories of artisanal fishers who have gained some (but still limited) access to consultation processes, marine ornamental fishers, which often are not even registered as fishers, so far have had no participation in decisions that directly affect their activity. As an example, none took part in the consultation process which led to the creation of the first regulatory measure for the marine fish ornamental trade in Brazil, the industry being solely represented by exporters of marine aquarium fish.

Controls and management of different forms of marine artisanal fisheries have traditionally been attempted (and in many cases not achieved) in Brazil through measures which often found little local social ressonance. With regards to the marine ornamental fisheries, no attempt to translate local knowledge into management has been made, even when fishers harvested seahorses within the boundaries of environmental protected areas. In that context, avenues for true participatory approaches dwindled, and words such as mistrust, misuse, misreporting, mis-management and misundertandings thrived.

Nevertheless, in the last few years the management of marine artisanal fisheries in Brazil has produced some interesting examples of species participatory management (e.g., land crabs), and concomitantly, more recent categories of protected areas in Brazil ("Reservas Extrativistas" and "Reservas de Desenvolvimento Sustentável") allow for grater community participation in resource management [60]. A similar approach can be sought after in conjunction with seahorse fishers, by coupling their knowledge with strategies such as temporal and/or spatial closure of fishing areas, and maintenance of harvested seahorses in the wild until selling the specimens.

Information provided by fishers clearly indicate the need to also address the issue of seahorse bycatch in the North and Northeastern regions of Brazil. Previously, focus has been directed to the SE-S regions of the country, where a second species of seahorse is caught in shrimp trawls, and sold as part of the dried trade. Also: recognition that seahorse bycatch is an important issue to seahorse conservation in Brazil will require new strategies on the part of the environmental agencies. So far, the focus of seahorse conservation in Brazil has been the live trade, which has been tentatively controlled through export quotas. Nevertheless, the information provided by fishers interviewed in this study indicate the need to urgently address the incidental capture of seahorses.

In summary, we consider the following aspects as positive for the conservation of seahorses and their habitats in Brazil: 1) fishers were willing to dialogue with researchers; 2) although capture and/or trade of brooding seahorses occurred, most interviewees recognized the importance of reproduction to the maintenance of seahorses in the wild (and therefore of their source of income), and expressed concern over population declines; 3) fishers associated the presence of a ventral pouch with reproduction in seahorses (regardless of them knowing which sex bears the pouch), and this may facilitate the construction of collaborative management options designed to eliminate captures of brooding specimens; 4) fishers recognized microhabitats of importance to the maintenance of seahorse wild populations; 5) fishers who kept seahorses in captivity tended to recognize the conditions as poor, and as being a cause of seahorse mortality.
